# A double diamond model-based approach to the innovative design of mobility scooters for the older adults

**DOI:** 10.3389/fpubh.2025.1672580

**Published:** 2025-12-02

**Authors:** Xianzhi Wang, Zefeng Zhao, Hang Yuan, Huijuan Ai

**Affiliations:** 1 Hubei Business College, School of Art and Communication, Wuhan, China; 2 Faculty of Fine and Applied Arts, Burapha University, Mueang Chonburi, Thailand

**Keywords:** older adults mobility, product innovation design methodology, double diamond model, user requirements, population aging

## Abstract

**Introduction:**

The global trend of population aging is irreversible, and the safety of older adults mobility has become an increasing concern. In response to the gap between rapidly growing market demand and limited academic research, this study proposes a design methodology for older adults mobility scooters guided by the four stages of the Double Diamond model.

**Methods:**

In the Discover stage, core user requirements were identified through user behavior observation and the Kano model. In the Define stage, multi-criteria decision-making (MCDM) was used to calculate the weight of each requirement. In the Develop stage, Quality Function Deployment (QFD) translated high-priority user needs into technical features and calculated their hierarchical weights. In the Deliver stage, these technical features were further translated into concrete design solutions.

**Results:**

This study provides a comprehensive and systematic framework for the innovative design of older adults mobility scooters. It aims to enhance the feasibility and design efficiency of older adults mobility products and contribute to addressing the challenges of population aging.

## 1 Introduction

The global aging population has become a critical and pressing issue, with far-reaching implications for healthcare systems, social structures, and economies (1). Studies show that the proportion of older adults individuals is steadily increasing worldwide, driven by declining birth rates and extended life expectancy (2). This demographic shift presents profound challenges, including a surge in age-related health conditions, such as musculoskeletal disorders, cardiovascular diseases, and cognitive decline (3). These issues not only reduce the mobility and independence of older adults individuals but also increase their risk of developing depression (4, 5).

The World Health Organization highlights that as individuals age, the decline in physical function becomes a major factor leading to the loss of independence and reduced quality of life among older adults (6). Addressing these challenges requires prioritizing the improvement of mobility in older adults populations. Mobility aids for older adults, such as mobility scooters, are essential in addressing this need.

However, due to the rapid growth of the market, mobility scooter manufacturers and designers often fail to meet the increasingly complex and evolving needs of older users (7). This has led to several critical issues that remain unresolved. First, complicated user interfaces and unfriendly controls severely limit the ability of older adults individuals to use these devices independently (8), thereby compromising their autonomy in daily life. Second, most current products lack adequate consideration of ergonomics, comfort, and safety (7), resulting in poor user experience during long-term use. More importantly, emotional care and social identity have long been overlooked in the design of these products (9). The appearance, materials, and functional configuration of many mobility scooters still exhibit a strong “medical device” esthetic (10), which fails to foster a sense of dignity or belonging for users (11). When used in public settings, these devices often lead to feelings of being stared at or stigmatized, further reinforcing the marginalization of older adults (12).

Therefore, mobility scooters should not only address physical needs but also convey care, respect, and esthetic value through design. A thoughtful design approach can help eliminate the psychological burden of social stigma among older users and promote their active participation and confident expression in society. This study aims to explore a new intelligent mobility scooter that integrates both human-centered care and technological rationality. Through innovative design language and multidimensional functional integration, we seek to provide the aging population with a dignified and emotionally supportive mobility solution.

To meet the growing demand for high-quality mobility scooters among older adults, it is crucial to adopt scientifically grounded design methods that enhance user satisfaction. Achieving user satisfaction among older adults populations involves multiple interrelated factors, making it a typical multi-criteria decision-making (MCDM) problem. MCDM methods have been widely validated as effective tools in diverse design applications (13–16). Among these, the Analytic Hierarchy Process (AHP) is one of the most established and widely applied approaches in MCDM. For example, Liu et al. used the AHP method to optimize the function and user experience of public electric vehicle charging stations (17). Similarly, Xie et al. applied AHP to guide the esthetic design of public facilities at Xiong’an Railway Station (18). Although AHP has been criticized for its subjectivity (19), several complementary techniques—particularly the Kano model (20) and Quality Function Deployment (QFD) (21)—have proven effective in mitigating these limitations. When initial product requirements are unclear, user journey mapping can help identify key pain points in current older adults mobility scooter use and extract core design elements (22). In the needs analysis stage, the Kano model assists designers in understanding how users evaluate product features and in prioritizing needs that most strongly impact satisfaction (23). In terms of evaluating design elements, AHP allows a structured analysis of the relative importance of each user requirement, offering clearer guidance to designers (24). Finally, in the product development stage, QFD can translate abstract requirements into actionable design parameters, ensuring the alignment of product features with user expectations (25).

The Double Diamond model, developed by the UK Design Council in 2004 (26), is a widely recognized framework for design thinking. Its core value lies in accurately identifying the right problem and delivering appropriate solutions. As a standardized methodology, it has been proven effective across a wide range of design projects (27). In this study, the application of the Double Diamond model provides a clear and structured approach to user-centered product development. Its use not only enhances methodological rigor but also strengthens the alignment between user insights and the final design outcomes.

Building upon these strengths, this study integrates the Double Diamond design model with a Multi-Criteria Decision-Making (MCDM) approach to establish a comprehensive design framework for older adults-oriented mobility scooters. This integrated framework not only accommodates the multifaceted and evolving needs of senior users but also provides actionable insights for designers aiming to develop functionally effective and user-centered assistive mobility solutions.

## 2 Materials and methods

This study primarily focuses on methodological research and does not involve human organs, animal tissues, or other living organisms. Human participation in this study is limited to behavioral observation and interview surveys focusing on older adults individuals in outdoor environments. Before the study commenced, the researchers informed all participants about the purpose of the research, the survey process, the use of the collected data, and the participants’ rights, obtaining their consent. All participants signed the informed consent form. Behavioral observations and interviews were conducted from May 10 to July 10, 2024. To ensure participants’ privacy, all data were anonymized so that no personal information could be identified. The research methods and procedures comply with ethical standards and regulations. This study was approved by the Ethics Review Committee of Hubei Business College. Ethics Approval Number: 202405043.

**Figure 1:**
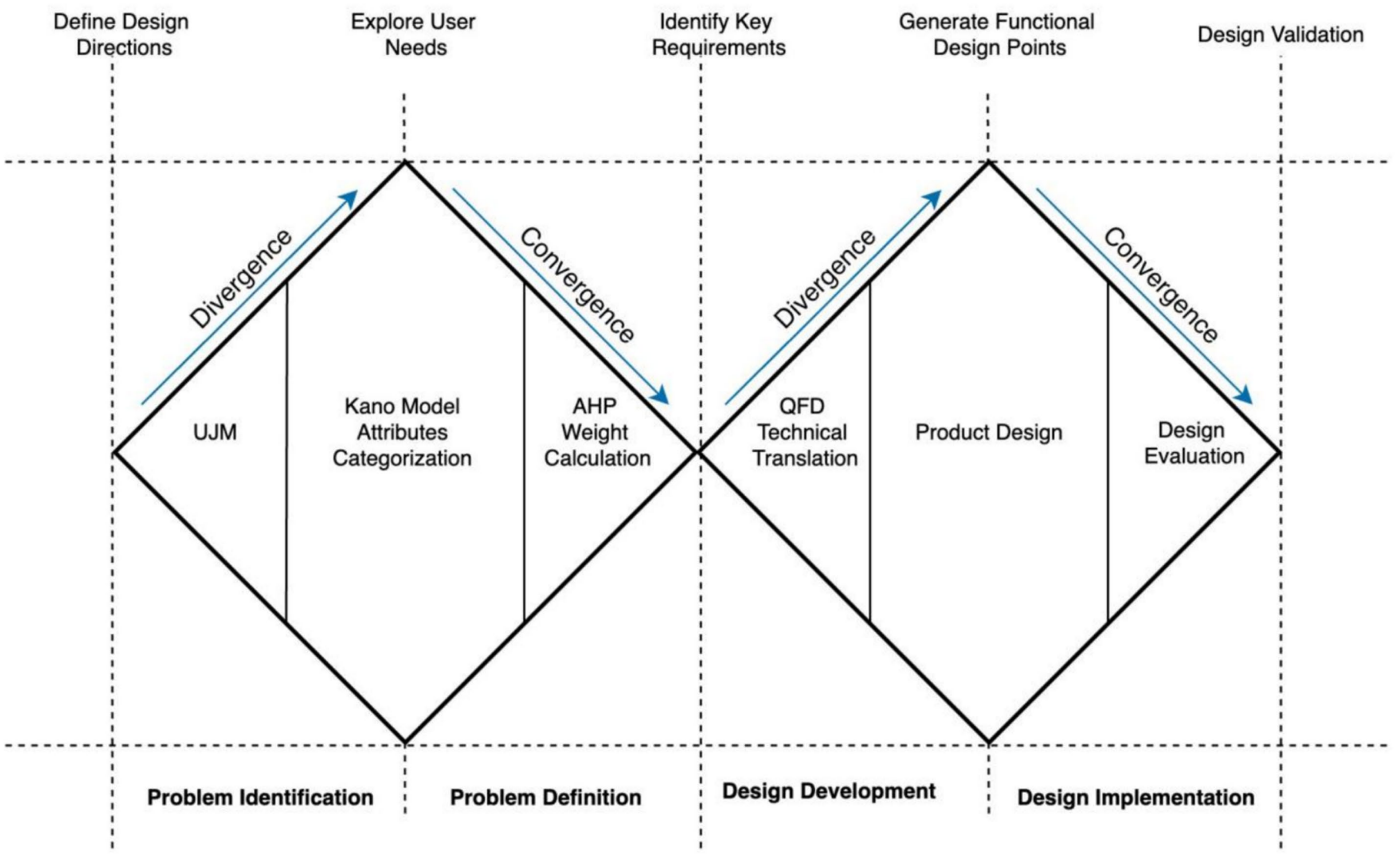
Design framework of older adults mobility scooter.

This study adopted the Double Diamond design model in combination with the user journey map to guide the innovative design of a mobility scooter for older adults users. The design process was divided into four key stages:

1. Problem Identifilcation: User behavior was observed to identify pain points in the daily use of mobility scooters by older adults. These insights were visualized to determine relevant design entry points.
2. Problem Definition: The Kano model was applied to categorize user needs into basic, performance, and attractive attributes. These needs were further prioritized using the Analytic Hierarchy Process (AHP) to identify those with the highest weights.
3. Design Development: The Quality Function Deployment (QFD) method was used to construct the House of Quality. This step translated prioritized user needs into specific technical features for product development.
4. Design Implementation: The final design phase focused on targeted product development guided by the technical indicators identified through QFD. The flowchart is shown in Figure 1.

## 3 Analysis and results

### 3.1 Older adults mobility scooter user needs

**Figure 2:**
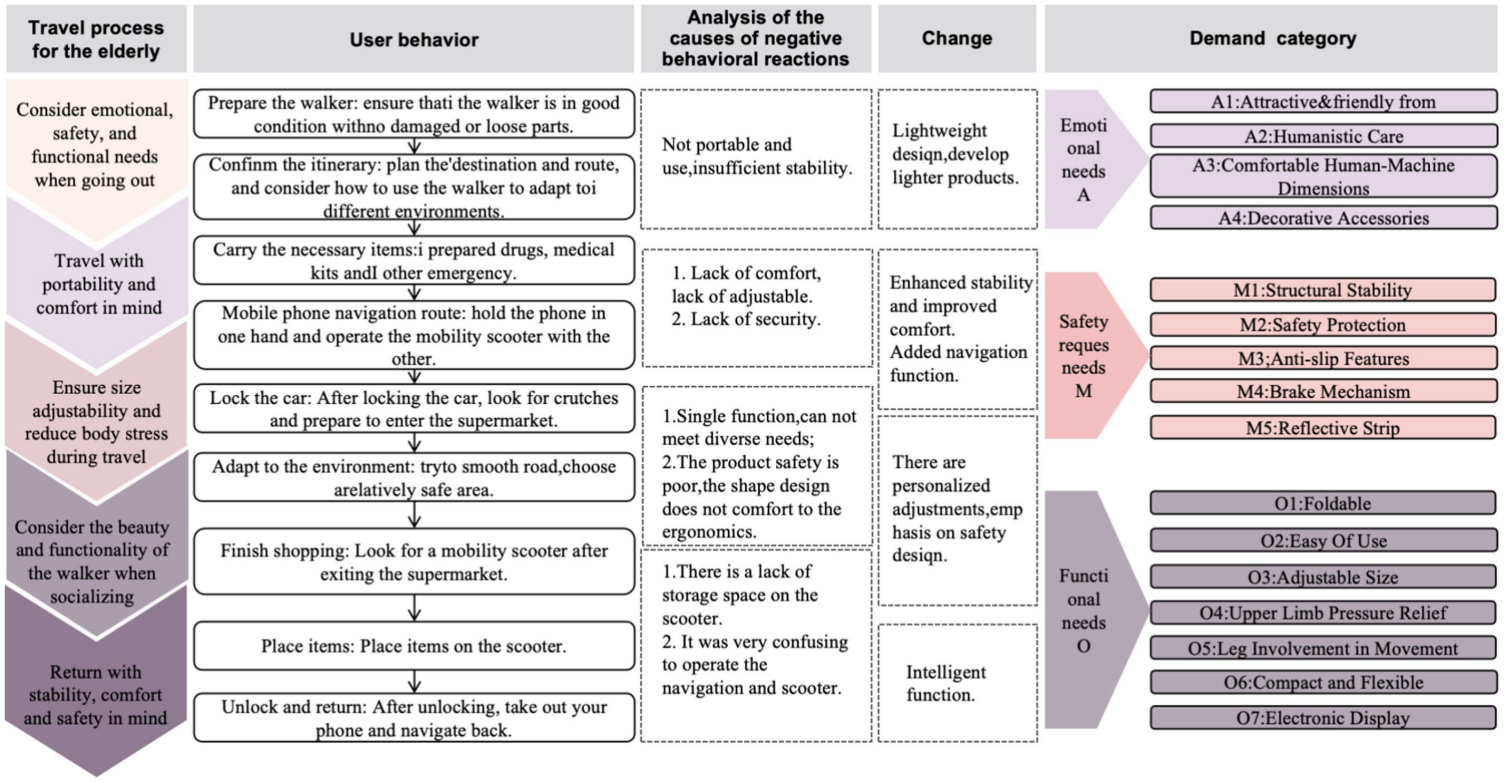
User journey map.

In this study, the Kano model is used to identify key user needs in the design of Older adults Mobility Scooters. The Kano model, developed by Japanese scholar Noriaki Kano, provides a comprehensive framework for understanding customer satisfaction through different categories of product features (28). It was further explored by Berger and colleagues, who highlighted its significance in quality management and customer satisfaction (29). This model has found wide applications across various industries, from airline services to leadership theory and even in areas such as radical innovation and political elections (30, 31). It has become a valuable tool in contemporary research, especially in product design and service optimization to enhance user satisfaction (32–34). To better understand the latent needs of older adults users, this study observed the complete shopping process of five individuals aged 65 and older who used mobility scooters to visit a supermarket. The focus was on identifying the challenges they encountered and their potential needs during the outing, as illustrated in Figure 2.

By analyzing the behavioral journey map of older adults users, we can identify opportunity points in the design of mobility scooters for seniors and categorize them into functional needs (O), safety needs (M), and emotional needs (A), as shown in Table 1.

**Table 1:** User needs categories.

Target demand	Demand category	Subordinate demand
Demands of older adults mobility aid users	Emotional needs A	A1: Attractive & friendly form
		A2: Humanistic Care
		A3: Comfortable Human-Machine Dimensions
		A4: Decorative Accessories
	Safety needs M	M1: Structural Stability
		M2: Safety Protection
		M3: Anti-slip Features
		M4: Brake Mechanism
		M5: Reflective Strips
	Functional needs O	O1: Foldability
		O2: Ease of Use
		O3: Adjustable Size
		O4: Upper Limb Pressure Relief
		O5: Storage space
		O6: Compact and Flexible
		O7: Electronic Display

### 3.2 KANO questionnaire design and analysis

This study uses the KANO model to classify user needs into five categories: Must-be Requirements (M), One-dimensional Requirements (O), Attractive Requirements (A), Indifferent Requirements (I), and Reverse Requirements (R) (35). The specific user needs for Older adults Mobility Scooter design are detailed in Table 2.

**Table 2:** User needs for mobility scooters.

Requirements attributes	Explanation details
Must-be requirements M	The basic requirements provided by the basic M-mobility scooter must be met, and if they are not met, the degree of satisfaction will decrease.
Attractive requirements A	Meets this need in the design of mobility scooter, the satisfaction will be improved, and vice versa.
Indifferent requirements I	The influence is not considered in the design of the mobility scooter.
Reverse type R	In the design of the mobility scooter, if it is insufficient, it will be reduced.
One-dimensional requirements O	Meets the hidden needs of users. If it is not met, it will not affect, but if it is met, it will be greatly improved.

The Kano questionnaire captures responses from both positive and negative perspectives. Positive questions represent satisfied needs, while negative questions address unmet ones. Each question offers five response options: Like (5), Must-be (4), Neutral (3), Live with (2), and Dislike (1). The demand attributes for each element are determined based on the evaluation criteria in Table 3.

**Table 3:** Comparison table of Kano model evaluation results classification.

Function/service		Negative problem				
		Dislike (1 point)	Live with (2 points)	Neutral (3 points)	Must-be (4 points)	Like (5 points)
Forward problem	Dislike (1 point)	Q	R	R	R	R
	Live (2 points)	M	I	I	I	R
	Neutral (3 points)	M	I	I	I	R
	Must-be (4 points)	M	I	I	I	R
	Like (5 points)	O	A	A	A	Q

Attractive (A), One-dimensional (O), Must-be (M), Indifferent (I), Reverse (R), and Questionable (Q).

The sample was drawn from a provincial capital in central China, where the distinct seasonal climate allows for better observation of older adults living habits from a climatic perspective. To identify key design elements for walking aids, questionnaires were distributed in three nursing homes, each housing over 50 residents. A preliminary questionnaire design is shown in Table 4, with the full version in Appendix S1. A total of 130 questionnaires were distributed, yielding 91 valid responses. Among the respondents, 72.5% were male, 27.5% were female, and ages ranged from 60 to 85 years.

**Table 4:** Kano questionnaire example.

Indicators	Question Setting
A1: Attractive and friendly form	How do you feel if A1 indicator appears in the design of mobility scooter? □ like □ accept □ do not care □ can live with it □ dislike
	How do you feel if A1 indicator does not appear in the mobility scooter design? □ like □ accept □ do not care □ can live with it □ dislike
A2: Humanistic care	How do you feel if A2 indicator appears in the design of mobility scooter? □ like □ accept □ do not care □ can live with it □ dislike
	How do you feel if A2 indicator does not appear in the mobility scooter design? □ like □ accept □ do not care □ can live with it □ dislike
A3: Comfortable human-machine dimensions	How do you feel if A3 indicator appears in the design of mobility scooter? □ like □ accept □ do not care □ can live with it □ dislike
	How do you feel if the A3 indicator does not appear in the mobility scooter design? □ like □ accept □ do not care □ can live with it □ dislike

To ensure the dependability of the questionnaire data for further analysis, a reliability assessment was conducted. Table 5 shows that the Cronbach’s *α* values for both positive and negative items exceed 0.8, indicating high reliability and suitability for subsequent calculations.

**Table 5:** Kano model questionnaire reliability results.

Problem Type	Sample size	Items	Cronbach’s α
Forward problem	91	16	0.843
Inverse problem	91	16	0.817

To assess the reasonableness of the questionnaire design and collected data, SPSS software was used for analysis. The KMO value was 0.776, and Bartlett’s test of sphericity produced an approximate chi-square value of 576.25, as shown in Table 6. These findings confirm the questionnaire’s high validity.

**Table 6:** Kano model questionnaire validity results.

KMO value		0.776
Bartlett sphericity test	Approximate chi-square	576.25
	df	186
	Sig	0.000

The Satisfaction Index (SI) and Dissatisfaction Index (DSI) are key metrics, where SI is typically positive and DSI is usually negative. Refer to Formulas 1, 2 for details.

$$\mathrm{SI}=\frac{A+O}{A+O+M+I}$$ (1)

$$\mathrm{DSI}=(-1)\cdot \frac{O+M}{A+O+M+I}$$ (2)

The Better-Worse coefficient reflects the selection ratio of each demand. A SI close to 1 indicates that fulfilling the demand increases user satisfaction, while a DSI near −1 suggests that not fulfilling the demand may enhance satisfaction. The analysis results of the Kano questionnaire user needs are shown in Table 7.

**Table 7:** Kano model analysis summary.

Functions/ Services	A	O	M	I	R	Q	Classification result	SI	DSI
A1	0%	31.25%	56.25%	12.5%	0%	0%	M	87.5%	−31.25%
A2	0%	43.75%	46.88%	9.38%	0%	0%	M	90.63%	−43.75%
A3	25%	56.25%	6.25%	12.5%	0%	0%	O	62.5%	−81.25%
A4	0%	5.88%	5.88%	35.29%	52.94%	0%	R	25%	−12.5%
M1	43.75%	46.88%	0%	9.38%	0%	0%	O	46.88%	−90.63%
M2	62.5%	34.38%	0%	3.13%	0%	0%	A	34.3%	−96.88%
M3	43.75%	40.63%	12.5%	3.13%	0%	0%	A	53.1%	−84.38%
M4	34.38%	43.75%	15.63%	6.25%	0%	0%	O	59.3%	−78.13%
M5	0%	0%	5.88%	35.29%	58.82%	0%	R	14.29%	0%
O1	9.38%	21.88%	56.25%	12.5%	0%	0%	M	78.1%	−31.25%
O2	15.63%	50%	28.13%	6.25%	0%	0%	O	78.1%	−65.63%
O3	15.63%	37.5%	28.13%	18.75%	0%	0%	M	65.6%	−53.13%
O4	25%	50%	15.63%	9.38%	0%	0%	O	65.63%	−75%
O5	12.5%	53.13%	25%	9.38%	0%	0%	O	78.13%	−65.63%
O6	9.38%	25%	40.63%	25%	0%	0%	M	65.63%	−34.38%
O7	0%	5.88%	5.88%	5.88%	82.35%	0%	R	66.67%	−33.33%

Attractive (A), One-dimensional (O), Must-be (M), Indifferent (I), Reverse (R), and Questionable (Q).

**Figure 3:**
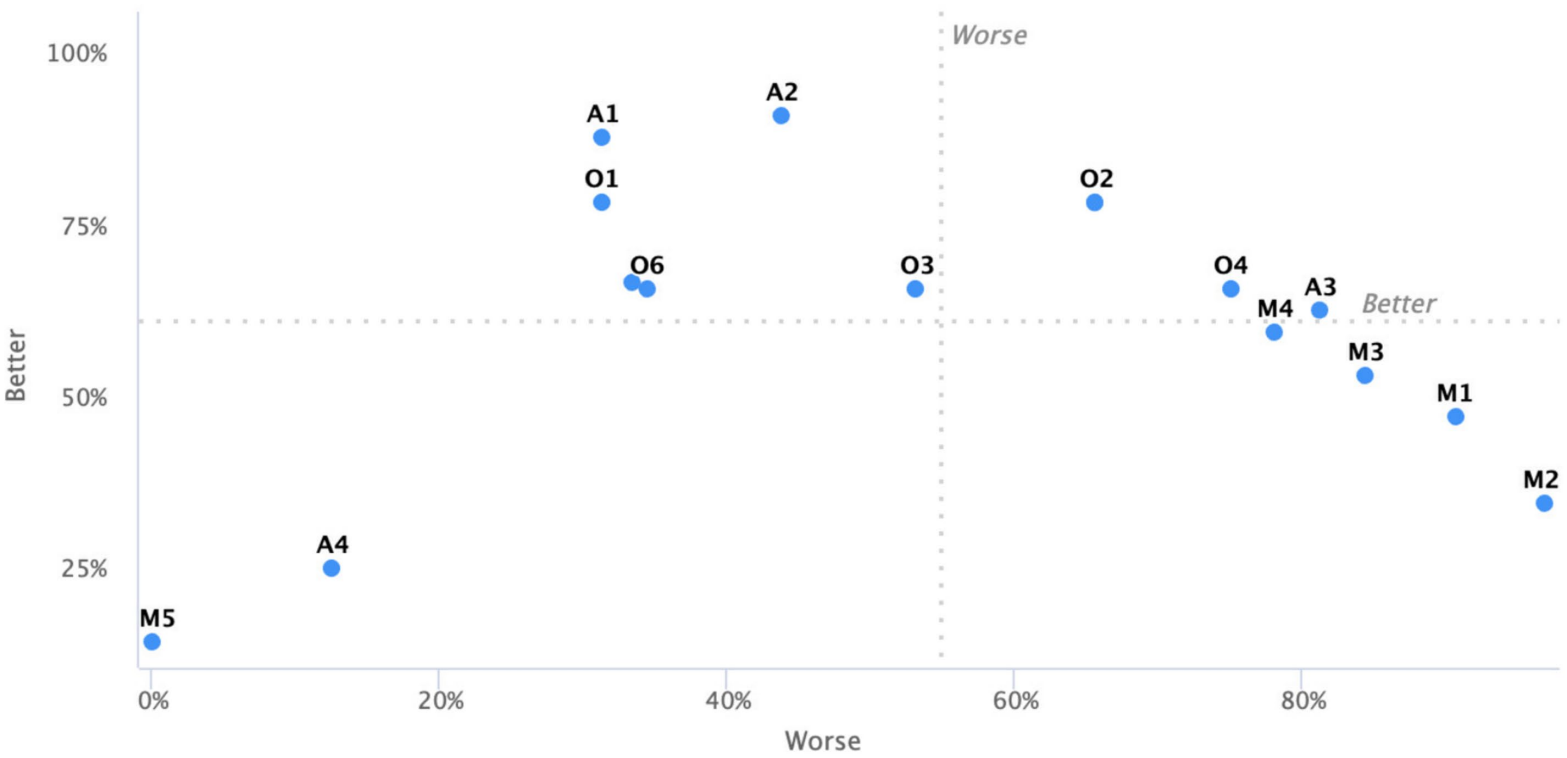
Better-worse coefficient analysis visualization.

The KANO model analysis highlights significant differences in how various needs affect user satisfaction. As shown Figure 3. Excitement Needs (e.g., easy operation, reducing upper limb strain, ergonomic dimensions) are critical for enhancing satisfaction and warrant focused innovation. Performance Needs (e.g., adjustable size, foldable design, compact structure, appealing esthetics) align closely with user expectations and require optimization. Basic Needs (e.g., structural stability, safety protection, anti-slip mechanisms) are essential and must be fully met, while Indifferent Needs (e.g., reflective strips, decorative accessories) have minimal impact and can be excluded.

### 3.3 User requirement analysis using the analytic hierarchy process (AHP)

In this study, Analytic Hierarchy Process (AHP) is used to establish and rank user requirement indicators, reducing design biases that may stem from subjective designer intentions. The Analytic Hierarchy Process (AHP) is a multi-criteria decision-making method introduced by Thomas L. Saaty in the 1970s (36). This method systematically integrates qualitative and quantitative analysis (37). AHP is especially effective for complex decision-making, helping decision-makers make well-informed choices based on multiple criteria (38, 39). Research has shown that AHP plays a crucial role in policymaking, project evaluation, business management, and strategic planning (39–41).

Based on the analysis of the Kano questionnaire, user satisfaction is primarily influenced by Basic Needs (M), Performance Needs (O), and Excitement Needs (A), whereas Indifferent Needs (I) have little impact. As a result, 13 out of 16 design elements were selected as key evaluation criteria. Subsequently, the Analytic Hierarchy Process (AHP) was employed to establish a hierarchical model with three levels: the Goal Layer, the Criteria Layer, and the Indicator Layer. Detailed information is presented in Table 8.

**Table 8:** Mobility scooter demand analysis model.

Target layer	Criterion layer	Indicator layer
Older adults walking aid products V	Emotional needs A	A1: Attractive and friendly form
		A2: Humanistic care
		A3: Comfortable human-machine dimensions
	Safety needs M	M1: Structural stability
		M2: Safety protection
		M3: Anti-slip features
		M4: Brake mechanism
	Functional needs O	O1: Foldability
		O2: Ease of use
		O3: Adjustable size
		O4: Adjustable safety guardrail
		O5: Flexible movement
		O6: Compact and lightweight

To enhance the rigor of the study, we assembled a review panel consisting of 10 experts and users. The panel includes: 2 full-time older adults care workers with 10 years of professional experience; 3 senior physical therapists; 2 designers specializing in medical assistive devices; 2 rehabilitation therapists with over 5 years of clinical experience; and 1 rehabilitation psychotherapy expert with more than 10 years of experience in older adults psychological and emotional therapy. The scoring criteria are detailed in Table 9, the weights of the criterion-level attributes are presented in Table 10, and the weights for secondary needs are shown in Tables 11–13.

**Table 9:** Saaty 1–9 scale.

Scale value	Importance Level	Implication
1	Coordinate with	Both are equally important.
3	A little more important	The former demand is slightly more important than the latter
5	Obviously more important	The former demand is obviously longer than the latter
7	Especially more important	The former demand is particularly more important than the latter
9	Extremely more important	The former demand is more important than the latter
2 4 6 8	——	Between the importance of the above median value

**Table 10:** Analysis of demand prioritization.

Target layer (V)	Functional needs O	Emotional needs A	Safety needs M	Weighted value
Functional needs O	1	4	2	0.57143
Emotional needs A	0.25	1	0.5	0.14286
Safety needs M	0.5	2	1	0.28571

**Table 11:** Emotional needs weight analysis.

Emotional needs A	A1	A2	A3	Weighted value
A1	1	3	1	0.42857
A2	0.333	1	0.333	0.14286
A3	1	3	1	0.42857

**Table 12:** Safety needs weight analysis.

Safety needs M	M1	M2	M3	M4	Weighted value
M1	1	2	4	4	0.50000
M2	0.500	1	2	2	0.25000
M3	0.250	0.500	1	1	0.12500
M4	0.250	0.500	1	1	0.12500

**Table 13:** Functional needs weight analysis.

Functional needs O	O1	O2	O3	O4	O5	O6	Weighted value
O1	1	0.2	0.500	0.111	0.125	2	0.04205
O2	5	1	3	0.500	0.500	8	0.19738
O3	2	0.333	1	0.250	0.250	3	0.07881
O4	9	2	4	1	2	9	0.36760
O5	8	2	4	0.500	1	9	0.28554
O6	0.500	0.125	0.333	0.111	0.111	1	0.02862

To ensure the reliability of the weights at the criterion and sub-criterion levels, consistency checks were performed using Formula 3. As shown in Table 14, all consistency ratios (CR) are below 0.1, confirming that the consistency requirements are satisfied.

$$\mathrm{CR}=\frac{\lambda_{\max}-n}{(n-1)\times \mathrm{RI}}$$ (3)

**Table 14:** Conformance test results.

Consistency check	The standard layer	M	O	A
CI	0.000	0.000	0.000	0.022
RI	0.520	0.520	0.890	1.260
CR	0.000	0.000	0.000	0.018

**Figure 4:**
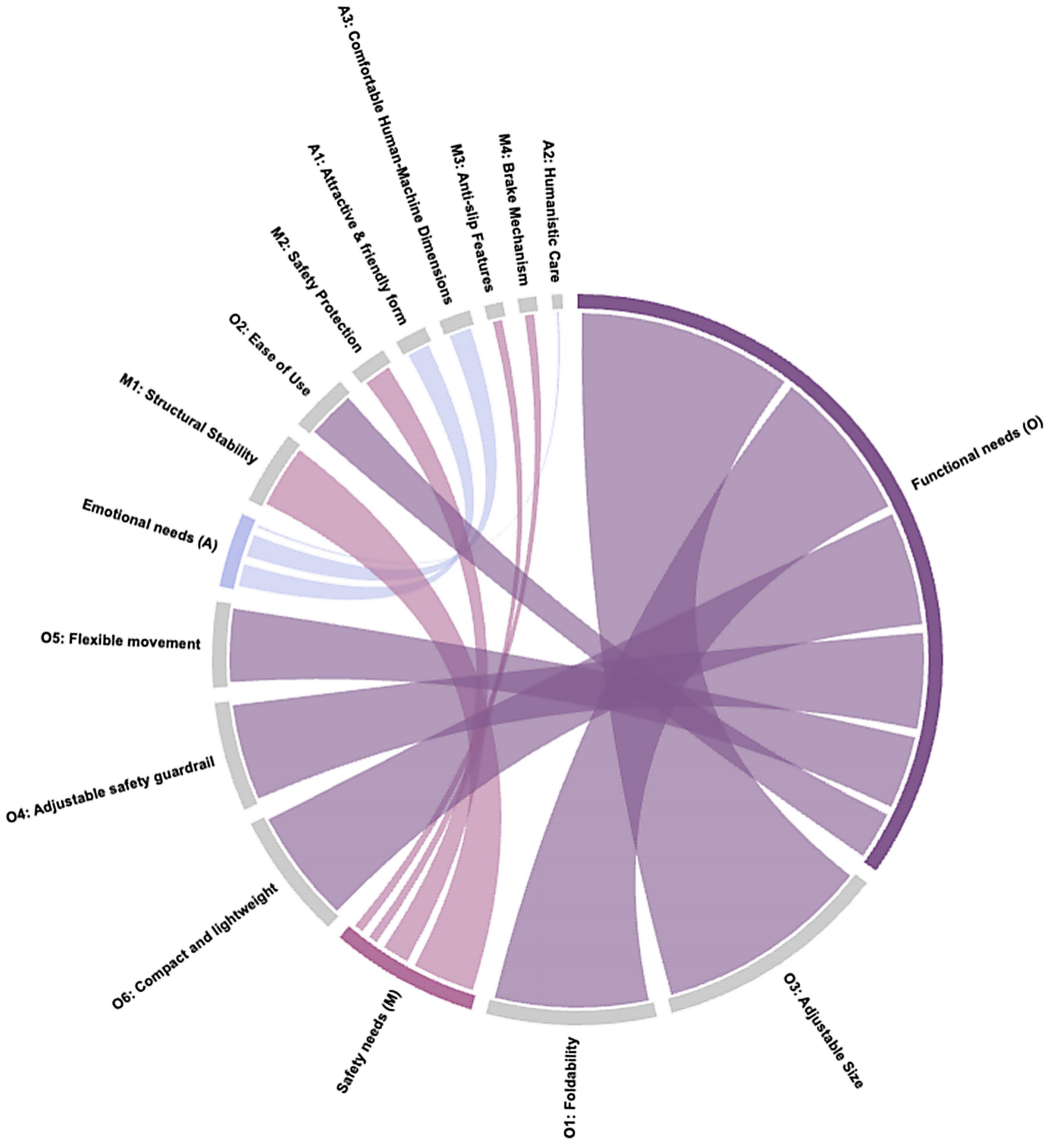
Chord diagram for weight values.

The resulting calculations are detailed in Table 15, and a chord diagram for the weight values is shown in Figure 4.

**Table 15:** Comprehensive weight ranking.

Demand type	Demand weight	Lower demand	Lower demand weight	Comprehensive weight value	Weight sorting
Emotional needs A	0.14286	A1: Attractive and friendly form	0.42857	0.0614	7
		A2: Humanistic care	0.14286	0.0204	12
		A3: Comfortable human-machine dimensions	0.42857	0.0614	6
Safety needs M	0.28571	M1: Structural stability	0.50000	0.1429	3
		M2: Safety protection	0.25000	0.0714	5
		M3: Anti-slip features	0.12500	0.0357	9
		M4: Brake mechanism	0.12500	0.0357	10
Functional needs O	0.57143	O1: Foldability	0.04205	0.0240	11
		O2: Ease of use	0.19738	0.1131	4
		O3: Adjustable size	0.07881	0.0450	8
		O4: Adjustable safety guardrail	0.36760	0.2098	1
		O5: Flexible movement	0.28554	0.1637	2
		O6: Compact and lightweight	0.02862	0.0164	13

By referencing the importance rankings in Table 15, designers can systematically prioritize key factors in the development of walking aids for the older adults, ensuring a more effective and user-centered design approach.

### 3.4 QFD indicator conversion

After calculating the user requirement weights for older adults mobility scooters using the Analytic Hierarchy Process (AHP), we applied Quality Function Deployment (QFD) to translate these requirements into technical specifications. The core of this process is the House of Quality (HOQ), which acts as a bridge between the “Voice of the Customer” and the “Voice of the Engineer” (42). The HOQ visually maps the relationship between user needs and product technical features. It also helps calculate the absolute and relative weights of these features, enabling the identification of potential issues in the design process. The HOQ construction involves the following steps:

Step 1: Construct the left wall of the HOQ.

Import the user requirements and their overall weights from Table 15 into the left wall of the House of Quality (HOQ), as shown in Table 16.

Step 2: Construct the roof of the HOQ.

**Table 16:** House of quality (HOQ) model.

Technical requirements/ User needs		User demand weight	Streamlined Design	Modular Component Design	Lightweight Vehicle Body	Multi-Directional Steering Wheels	Adjustable Safety Railings	Flexible Skin-Friendly Materials	Retractable Chassis	Anti-Slip Surface Texture Design	Warning Reflective Strip Design	Emergency Braking Device	Progressive Braking System	Foldable Walking Cane	Ergonomic Elastic Grip Design
Emotional needs	A1: Attractive & friendly form	0.0614	●	△		◎		◎	△	◎					◎
	A2: Humanistic Care	0.0204	◎		◎	△		◎			△			●	△
	A3: Comfortable Human-Machine Dimensions	0.0614	△				△		△						●
Safety needs	M1: Structural Stability	0.1429				●	◎								
	M2: Safety Protection	0.0714					●			●	●	△	△		
	M3: Anti-slip Features	0.0357				●		●		●		△	△		
	M4: Brake Mechanism	0.0357				△						●	●		
Functional needs	O1: Foldability	0.0240		△	△				△						
	O2: Ease of Use	0.1131	◎	●	△	△			△						
	O3: Adjustable Size	0.0450	◎	◎	◎									△	
	O4: Adjustable safety guardrail	0.2098					●								●
	O5: Flexible movement	0.1637		◎		●			△						
	O6: Compact and lightweight	0.0164	△		●									●	
Absolute weight			1.8098	4.0797	3.0141	2.2805	1.7331	0.2603	2.1747	0.5969	0.4182	0.4998	0.4998	2.5757	1.4786
Relative weights (%)			8.45%	19.05%	14.07%	10.65%	8.09%	1.22%	10.15%	2.79%	1.95%	2.33%	2.33%	12.02%	6.90%

Based on the technical indicators required to meet user needs, analyze and expand the technical specifications of the older adults mobility scooter (see Table 17).

Step 3: Summarize technical characteristics.

**Table 17:** Correspondence between user requirements and technical characteristics.

User primary needs	User secondary needs	Corresponding required skill indicators
Emotional needs (A)	A1: Attractive and friendly form	Streamlined design
		Modular component design
		Lightweight vehicle body
		Multi-directional steering wheels
	A2: Humanistic care	Adjustable safety railings
		Flexible skin-friendly materials
	A3: Comfortable human-machine dimensions	Adjustable safety railings
		Flexible skin-friendly materials
		Retractable chassis
Safety needs (M)	M1: Structural stability	Multi-directional steering wheels
		Anti-slip surface texture design
		Adjustable safety railings
		Retractable chassis
	M2: Safety protection	Warning reflective strip design
		Emergency braking device
	M3: Anti-slip features	Anti-slip surface texture design
		Flexible skin-friendly materials
	M4: Brake mechanism	Progressive braking system
		Anti-slip surface texture design
Functional needs (O)	O1: Foldability	Retractable chassis
		Foldable walking cane
	O2: Ease of use	Multi-directional steering wheels
		Retractable chassis
		Foldable walking cane
		Lightweight vehicle body
	O3: Adjustable size	Retractable chassis
		Adjustable safety railings
		Foldable walking cane
	O4: Adjustable safety guardrail	Ergonomic elastic grip design
		Adjustable safety railings
		Lightweight vehicle body
		Foldable walking cane
	O5: Flexible movement	Lightweight vehicle body
		Multi-directional steering wheels
	O6: Compact and lightweight	Lightweight vehicle body
		Retractable chassis
		Foldable walking cane

Refine and consolidate the results from Table 17 to generate a summary of the technical features of the older adults mobility scooter, as shown in Table 18.

Step 4: Import technical features into the HOQ.

**Table 18:** Product technical characteristics summary table.

No	Product technical features
1	User demand weight
2	Streamlined design
3	Modular component design
4	Lightweight vehicle body
5	Multi-directional steering wheels
6	Adjustable safety railings
7	Flexible skin-friendly materials
8	Retractable chassis
9	Anti-slip surface texture design
10	Warning reflective strip design
11	Emergency braking device
12	Progressive braking system
13	Foldable walking cane

Transfer the summarized technical features into the roof of the HOQ to establish the technical requirements.

Step 5: Construct the body of the HOQ.

Analyze the correlation between user needs and product technical features. Use ● (strong correlation), ◎ (moderate correlation), and △ (weak correlation) to indicate the strength of each relationship: ● = 5, ◎ = 3, △ = 1, and blank = 0. The correlation matrix is shown in Table 16.

Step 6: Construct the base of the HOQ.

Calculate the absolute and relative weights of each technical feature using Equations 4, 5. Import the results into the base of the HOQ, as presented in Table 16. The specific calculation formulas are as follows:

$$W_{j}=\sum_{i=1}^{q}W_{i}P_{\mathrm{ij}}$$ (4)

$$W_{k}=\frac{W_{j}}{\sum_{i=1}^{q}W_{j}}$$ (5)

In the formula:

$W_{j}$ ——Absolute weight of the quality characteristics of the older adults mobility scooter;

$W_{i}$ ——Comprehensive weight of user requirements;

$P_{\mathrm{ij}}$ ——Correlation coefficient between user requirements and quality characteristics;

$W_{k}$ ——Relative weight of the quality characteristics of the older adults mobility scooter.

Based on the above steps, the final House of Quality (HOQ) model for the product is obtained, as shown in Table 16.

Table 16 highlights that among all technical requirements, Modular Component Design, Lightweight Vehicle Body, and Adjustable Safety Railings obtained the highest relative weights, indicating their central roles in meeting older adults users’ needs for safety, comfort, and portability. In contrast, Flexible Skin-Friendly Materials, Warning Reflective Strip Design, and Anti-Slip Surface Texture Design exhibited comparatively lower weights, suggesting they serve as supplementary features to enhance user experience rather than core performance factors. Overall, the HOQ analysis quantitatively clarifies the hierarchy of design priorities and provides a data-driven foundation for the subsequent prototype development.

**Figure 5:**
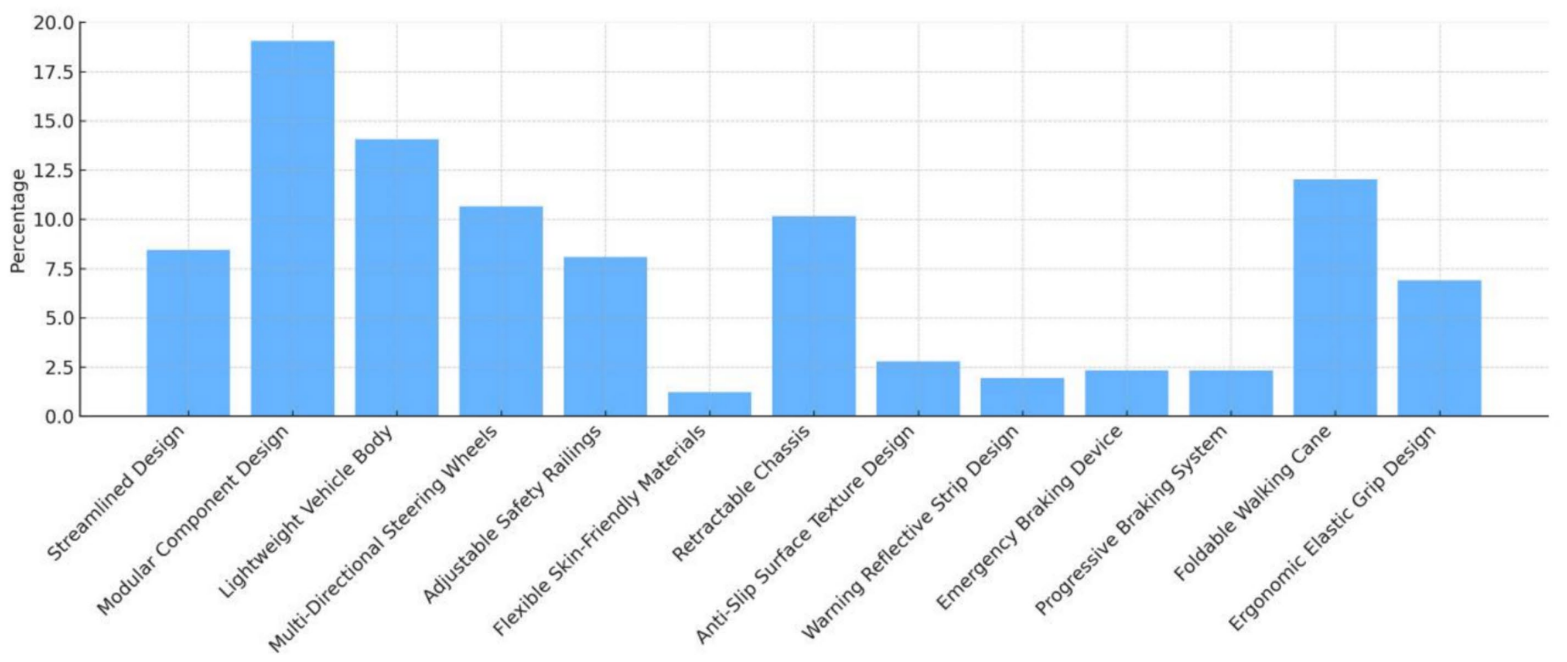
Design indicator weight description.

Based on the QFD (Quality Function Deployment) approach, a House of Quality model was developed to determine the relative importance of various design features, as shown in Figure 5. The analysis reveals that in the design of mobility scooters for older adults users, Modular Component Design (19.05), Lightweight Vehicle Body (14.07), Foldable Walking Cane (12.02), Multi-Directional Steering Wheels (10.65), and Retractable Chassis (10.15) received the highest importance weights. These features represent the most critical design priorities and should be given top consideration in subsequent product development. Following these, Streamlined Design (8.45), Adjustable Safety Railings (8.09), and Ergonomic Elastic Grip Design (6.90) also hold significant importance and should be optimized during the design process. In contrast, Flexible Skin-Friendly Materials (1.22), Warning Reflective Strip Design (1.95), Anti-Slip Surface Texture Design (2.79), Emergency Braking Device (2.33), and Progressive Braking System (2.33) are relatively less critical. While these features should not be ignored, they may receive comparatively lower priority when design resources are limited.

### 3.5 Discussion and result

**Figure 6:**
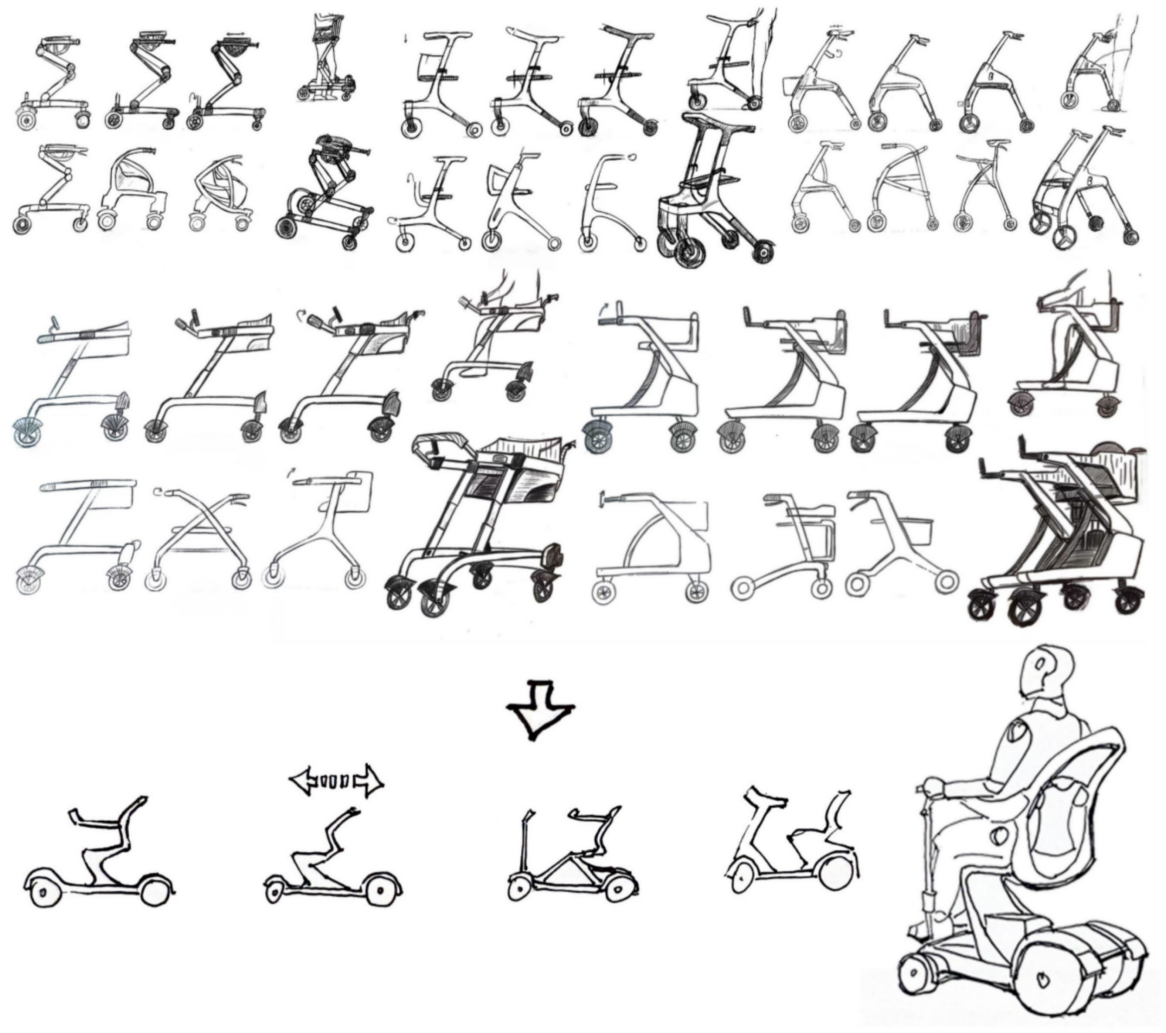
Scheme sketch design.

Based on the analyzed design elements of the older adults mobility scooter, an initial prototype design was developed, as shown in Figure 6.

**Figure 7:**
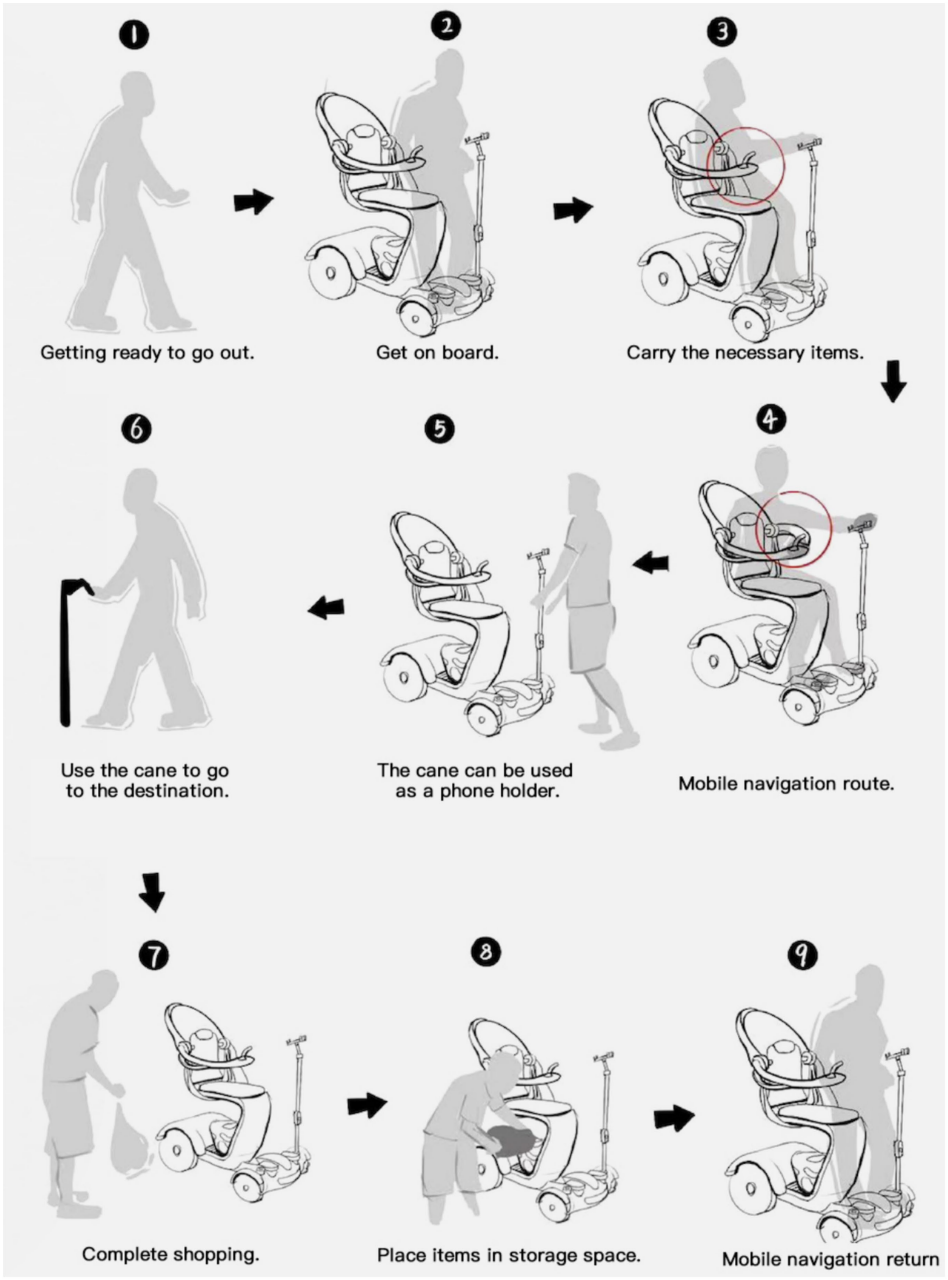
Product prototype.

Based on the problems identified through early-stage user behavior analysis, the designer proposed solutions through conceptual sketches and developed an experimental prototype accordingly. This process involved an immersive analysis of product usage scenarios to address real user pain points. The prototype is shown in Figure 7.

**Figure 8:**
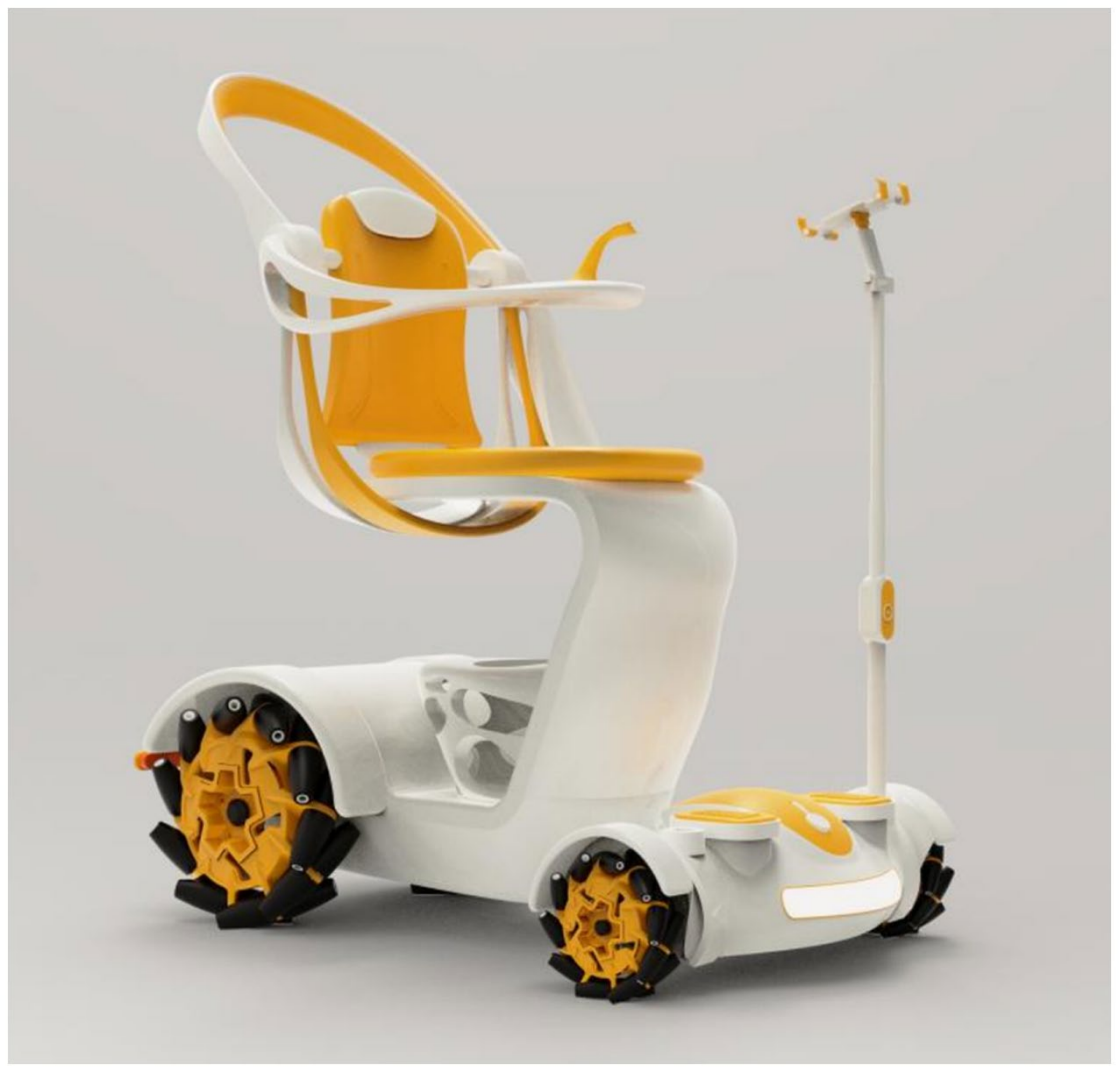
Final product prototype.

This electric mobility scooter emphasizes Modular Component Design, enhancing the product’s ease of use. The Lightweight Vehicle Body improves portability and ease of operation, making it especially suitable for older adults users with limited physical strength. The integration of a Foldable Walking Cane ensures continued mobility support even after dismounting, reflecting thoughtful consideration of real-life usage scenarios. In addition, Multi-Directional Steering Wheels significantly improve maneuverability in confined spaces, while the Retractable Chassis allows the scooter to adapt to various environments such as narrow corridors or storage areas. Other enhancements—including Streamlined Design, Adjustable Safety Railings, and Ergonomic Elastic Grip Design—further elevate overall safety, comfort, and user experience. As shown in Figure 8.

## 4 Conclusion

Due to the aging population, the demand for assistive products for the older adults is steadily increasing. However, traditional product designs often focus solely on basic functional requirements, while overlooking the emotional needs of older adults users and the complexity of their mobility-related demands. To address this gap, this study proposes an innovative mobility scooter design that prioritizes Modular Component Design, a Lightweight Vehicle Body, and an integrated Foldable Walking Cane to enhance the mobility experience of older adults. These user-centered design features collectively improve the overall quality of the mobility scooter and provide designers with targeted and practical design guidance.

Previous studies have shown that applying user-centered, multidimensional design decision-making methods in the design field can significantly enhance the feasibility of proposed solutions. For instance, Wu et al. utilized the Kano-AHP method to develop a quantifiable design approach for future smart jewelry (43). Cai et al. employed the KANO-AHP-FCE model to create culturally inspired products with an emphasis on emotional connections (44). However, these studies primarily focused on the weighting and evaluation stages, lacking an integrated framework that systematically guides the early discovery, definition, and iterative development of user needs.

Compared with previous studies, this research makes methodological improvements by embedding the Kano–AHP–QFD decision-making sequence within the Double Diamond design model. This integrated approach unifies user research, requirement prioritization, and design implementation into a coherent process, thereby enhancing methodological consistency and user-centered responsiveness. By combining exploratory qualitative analysis with quantitative evaluation, the study strengthens the scientific rigor of design decision-making and validates the effectiveness of the proposed framework through the development of an innovative product prototype.

Although this study has made some progress in uncovering latent user needs and improving the feasibility of decision-making, there are still certain limitations. For instance, during the user needs acquisition phase, the participants were older adults individuals from northern China. This sampling approach may lead to potential selection bias, as regional differences in living environments could result in variations in mobility habits, cultural preferences, and product use patterns among older adults. Additionally, most participants were recruited from nursing institutions rather than community settings, which may limit the generalizability of the findings to the broader older adults population. Furthermore, the sample size of observed participants was relatively small, and differences across age groups and body types could also influence users’ requirements for mobility scooters. Future research should expand the scope of the study to include older adults populations from diverse regions and living environments, allowing for a more comprehensive understanding of user diversity.

Moreover, the limited number of experts involved in the needs assessment may have influenced the precision of the results. Expanding the expert sample in future studies could ensure more balanced and reliable evaluations. In addition, this study did not include a large-scale sensitivity analysis of AHP weight variations due to resource constraints. Subsequent work will perform a detailed sensitivity analysis with a larger and more diverse sample to further verify the robustness and stability of the weighting results and strengthen the methodological reliability of the proposed framework.

## Data Availability

The original contributions presented in the study are included in the article/Supplementary material, further inquiries can be directed to the corresponding author/s.
