# Supplementary material for: A double diamond model-based approach to the innovative design of mobility scooters for the older adults
Source: Front Public Health. 2025 Dec 2;13:1672580. doi: 10.3389/fpubh.2025.1672580 (PMC12706668; doi:10.3389/fpubh.2025.1672580)
Supplement: Supplementary file 1 [file Table_1.DOCX]

**S1 Appendix：Elderly Mobility Scooters Design Kano Survey**

| Index | Question setting for two-way questioning (choose one answer for each question) |
| --- | --- |
| A1: Attractive & friendly form | How do you feel if A1 indicator appears in the design of walker?  □ like □ accept □ don't care □ can live with it□ dislike |
|  | How do you feel if A1 indicator doesn't appear in the walker design?  □ like □ accept □ don't care □ can live with it□ dislike |
| A2: Humanistic Care | How do you feel if A2 indicator appears in the design of walker?  □ like □ accept □ don't care □ can live with it□ dislike |
|  | How do you feel if A2 indicator doesn't appear in the walker design?  □ like □ accept □ don't care □ can live with it□ dislike |
| A3: Comfortable Human-Machine Dimensions | How do you feel if A3 indicator appears in the design of walker?  □ like □ accept □ don't care □ can live with it□ dislike |
|  | How do you feel if the A3 indicator doesn't appear in the walker design?  □ like □ accept □ don't care □ can live with it□ dislike |
| A4: Decorative Accessories | How do you feel if A4 indicator appears in the design of walker?  □ like □ accept □ don't care □ can live with it□ dislike |
|  | How do you feel if A4 indicator doesn't appear in the walker design?  □ like □ accept □ don't care □ can live with it□ dislike |
| M1: Structural Stability | How do you feel if M1 indicator appears in the design of walker?  □ like □ accept □ don't care □ can live with it□ dislike |
|  | How do you feel if M1 indicator doesn't appear in the walker design?  □ like □ accept □ don't care □ can live with it□ dislike |
| M2: Safety Protection | How do you feel if M2 indicator appears in the design of walker?  □ like □ accept □ don't care □ can live with it□ dislike |
|  | How do you feel if M2 indicator doesn't appear in the walker design?  □ like □ accept □ don't care □ can live with it□ dislike |
| M3: Anti-slip Features | How do you feel if M3 indicator appears in the design of walker?  □ like □ accept □ don't care □ can live with it□ dislike |
|  | How do you feel if the M3 logo doesn't appear in the walker design?  □ like □ accept □ don't care □ can live with it□ dislike |
| M4: Brake Mechanism | How do you feel if M4 indicator appears in the design of walker?  □ like □ accept □ don't care □ can live with it□ dislike |
|  | How do you feel if M4 indicator doesn't appear in the walker design?  □ like □ accept □ don't care □ can live with it□ dislike |
| M5: Reflective Strips | How do you feel if M5 indicator appears in the design of walker?  □ like □ accept □ don't care □ can live with it□ dislike |
|  | How do you feel if the M5 indicator doesn't appear in the walker design?  □ like □ accept □ don't care □ can live with it□ dislike |
| O1: Foldability | How do you feel if O1 indicator appears in the design of walker?  □ like □ accept □ don't care □ can live with it□ dislike |
|  | How do you feel if O1 indicator doesn't appear in the walker design?  □ like □ accept □ don't care □ can live with it□ dislike |
| O2: Ease of Use | How do you feel if O2 indicator appears in the design of walker?  □ like □ accept □ don't care □ can live with it□ dislike |
|  | How do you feel if O2 indicator doesn't appear in the walker design?  □ like □ accept □ don't care □ can live with it□ dislike |
| O3: Adjustable Size | How do you feel if O3 indicator appears in the design of walker?  □ like □ accept □ don't care □ can live with it□ dislike |
|  | How do you feel if O3 indicator doesn't appear in the walker design?  □ like □ accept □ don't care □ can live with it□ dislike |
| O4: Upper Limb Pressure Relief | How do you feel if O4 indicator appears in the design of walker?  □ like □ accept □ don't care □ can live with it□ dislike |
|  | How do you feel if O4 indicator doesn't appear in the walker design?  □ like □ accept □ don't care □ can live with it□ dislike |
| O5: Leg Involvement in Movement | How do you feel if O5 indicator appears in the design of walker?  □ like □ accept □ don't care □ can live with it□ dislike |
|  | How do you feel if O5 indicator doesn't appear in the walker design?  □ like □ accept □ don't care □ can live with it□ dislike |
| O6: Compact and Flexible | How do you feel if O6 indicator appears in the design of walker?  □ like □ accept □ don't care □ can live with it□ dislike |
|  | How do you feel if O6 indicator doesn't appear in the walker design?  □ like □ accept □ don't care □ can live with it□ dislike |
| O7: Electronic Display | How do you feel if O7 indicator appears in the design of walker?  □ like □ accept □ don't care □ can live with it□ dislike |
|  | How do you feel if O7 indicator doesn't appear in the walker design?  □ like □ accept □ don't care □ can live with it□ dislike |
