# Supplementary material for: A double diamond model-based approach to the innovative design of mobility scooters for the older adults
Source: Front Public Health. 2025 Dec 2;13:1672580. doi: 10.3389/fpubh.2025.1672580 (PMC12706668; doi:10.3389/fpubh.2025.1672580)
Supplement: Supplementary file 2 [file Table_2.DOCX]

**S2 Appendix：Fuzzy comprehensive evaluation calculation data of Scheme 2 and Scheme 3**

Scheme 1: The evaluation matrix of essential attributes is represented by $R_{A}$, expected attributes by $R_{M}$, and attractive attributes by $R_{O}$. The results are as follows

$$R_{A}=\left[ \begin{matrix} 0.4 & 0.3 & 0.1 & 0.1 & 0.1 \\ 0.3 & 0.4 & 0.2 & 0.1 & 0.0 \\ 0.1 & 0.3 & 0.3 & 0.2 & 0.1 \end{matrix} \right]$$

$$R_{M}=\left[ \begin{matrix} 0.5 & 0.2 & 0.2 & 0.1 & 0.0 \\ 0.4 & 0.3 & 0.2 & 0.1 & 0.0 \\ 0.1 & 0.3 & 0.3 & 0.2 & 0.1 \\ 0.3 & 0.1 & 0.2 & 0.3 & 0.1 \end{matrix} \right]$$

$$R_{O}=\left[ \begin{matrix} 0.0 & 0.2 & 0.2 & 0.2 & 0.4 \\ 0.3 & 0.3 & 0.2 & 0.1 & 0.0 \\ 0.1 & 0.3 & 0.3 & 0.3 & 0.0 \\ 0.1 & 0.4 & 0.3 & 0.2 & 0.0 \\ 0.2 & 0.4 & 0.2 & 0.1 & 0.1 \\ 0.2 & 0.4 & 0.4 & 0.0 & 0.0 \end{matrix} \right]$$

That is, the evaluation weight vector of scheme 1 is:

$$X_{A}=W_{A}\circ R_{A}=\left( 0.257 0.314 0.200 0.143 0.086 \right)$$

$$X_{M}=W_{M}\circ R_{M}=\left( 0.400 0.225 0.213 0.138 0.025 \right)$$

$$X_{O}=W_{O}\circ R_{O}=\left( 0.132 0.319 0.288 0.182 0.079 \right)$$

That is, the comprehensive evaluation weight matrix of scheme 1 is

$$P=W_{U}\circ X_{U}=W_{U}\circ\left[ \begin{matrix} X_{A} \\ X_{M} \\ X_{O} \end{matrix} \right]=\left( 0.226 0.291 0.254 0.164 0.065 \right)$$

Calculate the comprehensive percentage score of Scheme1:

$Y_{1}=P\circ V=$74.49

Scheme 3: The evaluation matrix of essential attributes is represented by $R_{A}$, expected attributes by $R_{M}$, and attractive attributes by $R_{O}$. The results are as follows

$$R_{A}=\left[ \begin{matrix} 0.6 & 0.3 & 0.1 & 0.0 & 0.0 \\ 0.5 & 0.3 & 0.1 & 0.1 & 0.0 \\ 0.6 & 0.4 & 0.0 & 0.0 & 0.0 \end{matrix} \right]$$

$$R_{M}=\left[ \begin{matrix} 0.6 & 0.3 & 0.1 & 0.0 & 0.0 \\ 0.7 & 0.2 & 0.1 & 0.0 & 0.0 \\ 0.7 & 0.2 & 0.1 & 0.0 & 0.0 \\ 0.6 & 0.1 & 0.2 & 0.1 & 0.0 \end{matrix} \right]$$

$$R_{O}=\left[ \begin{matrix} 0.4 & 0.2 & 0.2 & 0.1 & 0.1 \\ 0.2 & 0.4 & 0.4 & 0.0 & 0.0 \\ 0.4 & 0.4 & 0.2 & 0.0 & 0.0 \\ 0.7 & 0.1 & 0.1 & 0.1 & 0.0 \\ 0.6 & 0.2 & 0.1 & 0.1 & 0.0 \\ 0.7 & 0.3 & 0.0 & 0.0 & 0.0 \end{matrix} \right]$$

That is, the evaluation weight vector of scheme 1 is:

$$X_{A}=W_{A}\circ R_{A}=\left( 0.586 0.343 0.057 0.014 0.000 \right)$$

$$X_{M}=W_{M}\circ R_{M}=\left( 0.638 0.238 0.113 0.013 0.000 \right)$$

$$X_{O}=W_{O}\circ R_{O}=\left( 0.503 0.282 0.155 0.043 0.017 \right)$$

That is, the comprehensive evaluation weight matrix of scheme 1 is

$$P=W_{U}\circ X_{U}=W_{U}\circ\left[ \begin{matrix} X_{A} \\ X_{M} \\ X_{O} \end{matrix} \right]=\left( 0.553 0.278 0.129 0.030 0.010 \right)$$

Calculate the comprehensive percentage score of Scheme1:

$Y_{1}=P\circ V=$83.34
